# Supplementary material for: Mimicking Collective Firing Patterns of Hundreds of Connected Neurons using a Single-Neuron Experiment
Source: Front Neurosci. 2016 Jan 20;9:508. doi: 10.3389/fnins.2015.00508 (PMC4718983; doi:10.3389/fnins.2015.00508)
Supplement: Supplementary file 2 [file DataSheet1.pdf]

Appendix A

Experimental scheme: Discrete time, above-threshold connections (e.g. Figure 5A):

A mimicked network consists of N neurons. Each neuron has K randomly selected pre- and post- above-threshold synaptic connections, and all of their delays are τ. Additional external noise with a rate of F_{noise} was given. Typical values are K=[2, 10], F_{noise}=[0.5, 1.5] Hz, and τ=[10, 20] ms. The stimulation sequence for the mimicking neuron scheme consists of the last M stimulations given to the neuron. M is determined by the neuron's short-term memory, where typically M=3.

The initial conditions of stimulation times were generated for each neuron independently, by randomly choosing M+1 sorted integers in the range [2-round((M+1)/(2$\tau \cdot f_c$)),1], where f_c is the critical frequency of the neuron. The random numbers represent the stimulation times in the history of the neuron preceding the experiment, in the form of multiples of τ. Accordingly, the scheme starts at time τ and the first neurons that are stimulated are those whose maximal integer equals 1.

In **Figure 5A**, the values were N=500, K=2, F_{noise}=1 Hz, M=3 and τ=13 ms. The mimicking neuron had f_c=5 Hz, and was given 1500 pre-scheme stimulations with a rate of $2f_c$ to reach the intermittent phase.

The mimicking scheme is done using the following procedure (a simplified flowchart is presented in **Figure 4**):

Parameters:

N - Number of neurons.

K - Number of pre- and post-synaptic sub-threshold connections.

M - The number of stimulations in the stimulation sequence (the neuron's "memory").

F_{noise} - Noise frequency.

τ - The delay time between connected neurons.

f_c - The critical frequency of the mimicking neuron.

Edges - A **N**×**K** connectivity matrix.

Variables:

Queue1, **Queue2** - An empty 1×**N** array.

StimulationsData - A **N**×(**M**+1) matrix that holds the stimulation times.

Counter1, **Counter2** - A 1×**N** empty array.

SnapshotNo. - An integer denoting the currently mimicked snapshot.

1. Load connectivity to **Edges** and initial conditions to **StimulationsData**. **StimulationsData(n**,**m**) indicates a stimulation to neuron **n** at snapshot number **StimulationsData(n**,**m**).
   Assign **SnapshotNo.**=1.
   Insert to **Queue1** all the neurons that are stimulated at **SnapshotNo.**=1, according to the initial conditions (the maximal random integer is 1, i.e. the stimulation time equals **τ**).
2. For each neuron **n** in **Queue1**:
   - The mimicking neuron is stimulated **M** times, with the **M** corresponding last inter-stimulation intervals of neuron **n**, from **StimulationsData**(**n**).
   - Another stimulation is given after the appropriate inter-stimulation-interval. This stimulation will be also the first stimulation for the next mimicking process.
   - If the stimulation results in an evoked spike:
     Add the row **Edges**(**n**) to **Queue2**, without repetitions.
   - Replace the minimal value of **StimulationsData**(**n**) with **SnapshotNo.**.
3. Generate noise in **Queue2**: Add random neurons to **Queue2** with a probability of $\mathbf{F_{noise}\tau/N}$ for each neuron.
4. Clear **Queue1**, move **Queue2** into **Queue1** and clear **Queue2**.
5. Increment **Snapshot No.** by 1 and Go to clause 2.

Appendix B

Experimental scheme: Discrete time, sub-threshold connections (e.g. Figure 5B): This scheme is similar to the scheme presented in **Appendix A**, but also incorporates sub-threshold synaptic connections.

All synaptic connections are sub-threshold, and typically the number of postsynaptic connections per neuron, K, is between 10 and 50. K_{min} denotes the minimal number of simultaneous sub-threshold stimulations that can result in an evoked spike, typically between 2 and 5. During the procedure if the number of pre-synaptic neurons that fired in the previous snapshot is above K_{min}, the stimulation is above-threshold.

Above-threshold stimulation has a strength of -800 mV, with a duration of 200 μs. The strength of a sub-threshold stimulation is in the range of [-200, -500] mV with a duration of 200 μs. The memory of the stimulations accounts also for sub-threshold stimulations, where typically M=8. Initial conditions of stimulations are generated randomly similar to the first scheme, with additional below-threshold stimulations.

In **Figure 5B**, the values are N=500, K=50, K_{min}=4, F_{noise}=1 Hz, M=8, and τ=15 ms. The strength of the sub-threshold stimulation was -300 mV. The mimicking neuron had f_c=2.1 Hz, and was given 1000 pre-scheme stimulations with a rate of $2f_c$ to reach the intermittent phase.

The mimicking scheme is done using the following procedure:

Parameters:

N - Number of neurons.

K - Number of pre- and post-synaptic sub-threshold connections.

M - The number of stimulations in the stimulation sequence (the neuron's "memory").

$\mathbf{K_{min}}$ - The number of minimal concurrent stimulations that would result in an above threshold excitatory response.

$\mathbf{F_{noise}}$ - Noise frequency.

$\boldsymbol{\tau}$ - The delay time between connected neurons.

$\mathbf{f_c}$ - The critical frequency of the mimicking neuron.

Edges - A **N**×**K** connectivity matrix.

Variables:

Queue1, **Queue2** - An empty 1×**N** array.

StimulationsData - A **N**×(**M**+1)×2 matrix that holds the stimulation times and strengths.

Counter1, **Counter2** - A 1×**N** empty array.

SnapshotNo. - An integer denoting the currently mimicked snapshot.

1. As in **Appendix A**.
2. For each neuron **n** in **Queue1**:

   If **Counter1**(**n**)>=$\mathbf{K_{min}}$, the stimulation is above-threshold:

   - The mimicking neuron is stimulated **M** times, with the **M** corresponding inter-stimulation-intervals.
   - Above-threshold stimulation is given after the appropriate inter-stimulation-interval. This stimulation will be also the first stimulation for the next mimicking process.
   - If the stimulation results in an evoked spike:

     For each neuron **m** from the row **Edges**(**n**), add **m** to **Queue2**, if m already exists in **Queue2**, add 1 to **Counter2**(**m**).

   Replace the stimulation with the minimal time value in **StimulationsData**(**n**) with the time value of **SnapshotNo.** and the appropriate strength value.
3. Generate noise in **Queue2**: Add random neurons to **Queue2** with a probability of $\mathbf{F_{noise}\tau/N}$ for each neuron, and add $\mathbf{K_{min}}$ to **Counter2** accordingly.
4. Clear **Queue1**, move **Queue2** into **Queue1** and Clear **Queue2**.

   Clear **Counter1**, move **Counter2** into **Counter1** and Clear **Counter2**.
5. Increment **SnapshotNo.** by 1 and Go to clause 2.

Appendix C

Experimental scheme: Continues time, above-threshold connections (e.g. Figure 5C):

A continuous time version of **Appendix A**. The continuity of this scheme is limited by the machine cycle, 20 μs in our implementation. All synaptic connections are above-threshold, where typically K=2 and M=3. For each connection, τ was chosen randomly from [τ_{min}, τ_{max}], where typically τ_{min} is between 8 and 12 ms and τ_{max} is between 12 and 20 ms. Initial conditions are constructed by choosing M random delays using exponential distribution with a rate of $2f_c$.

In **Figure 5C**, the values are N=500, K=2, F_{noise}=0.5 Hz, M=3, τ_{min}=8 and τ_{max}=12 ms. The mimicking neuron had f_c=3 Hz, and was given 700 pre-scheme stimulations with rate of $2f_c$ to reach the intermittent phase.

The mimicking scheme is done using the following procedure:

Parameters:

N - Number of neurons.

K - The number of pre- and post-synaptic above-threshold connections.

M - The number of stimulations in the stimulation sequence (the neuron's "memory").

F_{noise} - Noise frequency.

f_c - The critical frequency of the mimicking neuron.

τ_{min}, τ_{max} - The time range of the delays.

Edges - A **N**×**K** matrix containing information of nodal connections and their delays. The delays are randomly chosen in the range [**τ_{min}, τ_{max}**].

Variables:

StimulationsData - A **N**×30 matrix that holds the stimulation times.

T - Mimicked time.

Counter - A 1×**N** array of zeros.

1. Load connectivity to **Edges** and initial conditions to **StimulationsData**.
   Assign **T**=0.

2. Find a neuron **n** such that **StimulationsData**(**n**,**Counter**(**n**)) is minimal but also greater than **T**. If no neuron is found - go to clause 4.
   Assign **T**=**StimulationsData**(**n**,**Counter**(**n**)).
3. The mimicking neuron is stimulated **M** times, with the **M** corresponding inter-stimulation-intervals of **n** (taken from **StimulationsData**).
   Another stimulation is given. This stimulation will be also the first stimulation for the next mimicking process.
   If the stimulations results in an evoked spike:
      For each neuron **j** from the row **Edges**(**n**), add the sum of the spike's time and neuron **j**'s delay to **StimulationsData**(**j**,**Counter**(**j**)).
   Increment **Counter**(**n**) by 1, assign **Counter**(**n**)=**Counter**(**n**) mod **N**.
4. Generate noise in **StimulationsData**: A stimulation every 0.1 ms with a probability of $10^{-4*}\mathbf{F_{noise}}$ to each neuron.
5. Go to clause 2.

Appendix D

Experimental scheme: Continuous time, Sub-Threshold connections (e.g. Figure 6):

A continuous time version of **Appendix B**. In the end of each mimicking process, if the last stimulation results in an evoked spike, a weak stimulations will be given to each one of the post synaptic neurons of the mimicked neuron. If two weak stimulations arrive with a short lime-lag between them, they will be merged to one strong stimulation. The procedure could be generalized to account for stricter temporal summations, where more than two weak stimulations are required to fall within a short time window in order to yield a spike (K_{min} can be defined similarly to **Appendix B**) - the current implementation requires only two stimulations. The program advances in segments of time with the size of SegmentLength, such that neurons whose current segment does not contain any above-threshold stimulations will not be mimicked at the current step. A stimulation sequence consists of the short term stimulation history of a neuron, [T - LookBack, T], where T is the time of the current time mimicked and LookBack is a pre-determined time constant. There is an essential difference between this procedure and all the other procedures that should be stressed out. In the previous procedures the times of the last M stimulations were taken into account, disregarding the length of the inter-stimulation-intervals. This procedure, on the other hand, takes into account all and only the stimulations that occurred within the last LookBack seconds.

Initial conditions are random times in the range [-LookBack-τ_{max}, 0], corresponding to the stimulation times. Only a third of the stimulations are above threshold stimulations. The average stimulation rate is $3f_c$.

In **Figure 6**, the values are N=350, K=13, NoiseNum=3, SegmentLength=4.5 ms, LookBack=3.5 s, MinGap=5.5 ms, τ_{min}=6 ms and τ_{max}=10 ms. The mimicking neuron had f_c=4.2 Hz, and was given 700 pre-scheme stimulations with a rate of $2f_c$ to reach the intermittent phase.

The mimicking scheme is done using the following procedure:

Parameters:

N - Number of neurons.

K - The number of pre- and post-synaptic sub-threshold connections.

$\mathbf{f_c}$ - The critical frequency of the mimicking neuron.

$\boldsymbol{\tau_{min}}$, $\boldsymbol{\tau_{max}}$ - The time range of the delays.

SegmentLength - The time segment in which the program advances, has to be smaller than the minimal neuronal response latency.

NoiseNum - The number of random neurons stimulated at every segment.

LookBack - The length of the mimicking sequence.

MinGap - The maximal time-lag between two weak stimulations which results in one merged supra-threshold stimulation.

Edges - **N**×**K** matrix, containing information of nodal connections and their delays. The delays are randomly chosen in the range [$\boldsymbol{\tau_{min}}$, $\boldsymbol{\tau_{max}}$].

Variables:

StimulationsData - A data structure that holds the times of weak and strong stimulations.

SegmentIndex - An index indicating the current segment (the time step being mimicked).

IsStimulated - **N**×**m** matrix, the first index indicates a neuron in the network and the second index indicates a Segment Index. **IsStimulated**(i,**SegmentIndex**)=1 means that the neuron is stimulated above-threshold at the segment Index **SegmentIndex**, otherwise it is not.

Next - a FIFO with the neurons that should be stimulated at the current segment.

1. Load connectivity to **Edges** and initial condition to **StimulationsData** and **IsStimulated**.
   Assign **SegmentIndex=** -1.
2. **SegmentIndex**=**SegmentIndex**+1.
   Add to **Next** all neurons **i**, such that **IsStimulated** (**i**,**SegmentIndex**)=1.
   Generate random above-threshold stimulations according to **NoiseNum**.
3. For each neuron **i** in **Next**:

- Wait several milliseconds.

- Stimulate the mimicking neuron with all the stimulations of **i** in the time range [(**SegmentIndex***SegmentLength*-**LookBack**), **SegmentIndex***SegmentLength*].

- If a spike occurred after the mimicking stimulation sequence:

    - Update **StimulationsData** for the post synaptic neurons of the mimicked neuron to receive a weak stimulation at time **SegmentIndex*****SegmentLength**+**L**+$\mathbf{D_{ik}}$, where **L** is the latency between the end of the mimicking stimulation sequence and the evoked spike, and $\mathbf{D_{ik}}$ is the delay between the mimicked neuron, **i**, and the postsynaptic neuron, **k**.

    - If a weak stimulation falls in the vicinity (i.e. time gap is smaller than **MinGap**) of an existing weak stimulation

        - Merge the two weak stimulations into one strong stimulation, and set the time of the strong stimulation to be the mean time of the two weak stimulations.

        - Update **IsStimulated** (**k**, **SegmentIndex_Stim**)=1, with **SegmentIndex_Stim** being the corresponding **SegmentIndex** of the strong stimulation time.

4. Go back to clause 2.
